# Tumeur stromale rectale: à propos d'une observation

**DOI:** 10.11604/pamj.2014.17.119.2285

**Published:** 2014-02-18

**Authors:** Haitham Rejab, Wala Ben Kridis, Hazem Ben Ameur, Jihene Feki, Mounir Frikha, Mohamed Issam Beyrouti

**Affiliations:** 1 Service de chirurgie générale, CHU Habib Bourguiba 3029 Sfax, Tunisie; 2 Service de carcinologie médicale, CHU Habib Bourguiba 3029 Sfax, Tunisie

**Keywords:** Rectum, tumeur stromale, echo endoscopie, immunohistochimie, traitement, Rectum, stromal tumor, echo endoscopy, immunohistochemistry, treatment

## Abstract

Les tumeurs stromales gastro-intestinales sont des tumeurs mésenchymateuses peu fréquentes. Elles sont localisées préférentiellement eu niveau de l'estomac. La localisation rectale reste rare. A un nouveau cas de tumeur stromale du rectum ainsi qu'une bref revue de la littérature, on se propose d’étudier les particularités cliniques, radiologiques et thérapeutiques de cette entité rare.

## Introduction

Les tumeurs stromales gastro-intestinales (GIST) sont des tumeurs mésenchymateuses peu fréquentes. Elles siègent le plus souvent au niveau de l'estomac (50-70%) et de l'intestin gêle (20-30%). La localisation rectale est extrêmement rare; elle représente seulement 5% des cas et 0.1% de toutes les tumeurs rectales [1, 2] A travers une nouvelle observation de tumeur stromale du rectum ainsi qu'un bref revue de la littérature, on se propose d’étudier les particularités anatomocliniques, radiologiques et thérapeutiques de cette entité rare.

## Patient et observation

**Figure 1:**
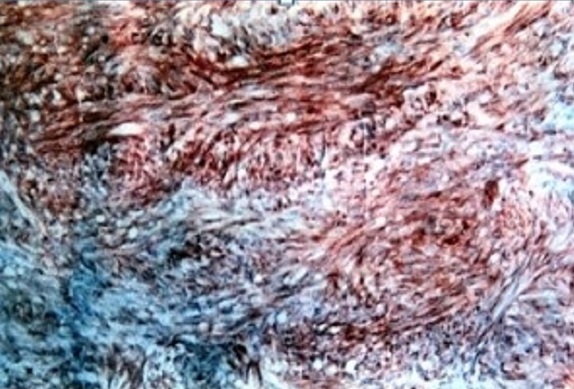
Marquage immuno-histochimique par l'anticorps C-Kit (CD 117)

**Figure 2:**
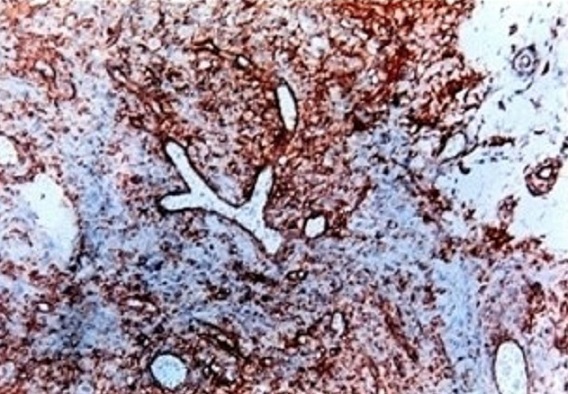
Marquage immuno-histochimique par l'anticorps CD 34

Une femme âgée de 63 ans, sans antécédents pathologiques particuliers, avait présenté des rectorragies associées à un syndrome rectal fait de ténesmes et épreintes. Le toucher rectal avait montré une masse bourgeonnante hémi circonférentielle; fixe et saignant au contacte, siégeant siégeant à 5 cm de la marge anale. Le reste de l'examen clinique était sans particularité. La rectoscopie avait conclu à la présence d'une masse tumorale hémi circonférentielle, ulcéro-bourgeonnante fixe par rapport au plan profond et superficiel, saignante au contact du rectoscope, faisant 2 cm de taille et située à 5 cm de la marge anale. La colonoscopie n'avait pas revélée d'autres lésions du cadre colique. L’étude anatomopathologique de la pièce de biopsie avait conclu à une prolifération d'aspect lobulée faite de cellules fusiformes ou épithéloïdes à cytoplasme peu abondant mal délimité et à noyau ovulaire ou allongé volumineux nucléolé à chromatine hétérogène; les atypies sont souvent modérées et les mitoses sont très nombreuses. Le complément d’étude immuno- histochimique avait montré que les cellules tumorales étaient positives pour vimentine, CD34, C-KIT et NSE, et sont négatives pour le reste des anticorps (Figure 1, Figure 2). Cet aspect est en faveur d'une tumeur stromale maligne du bas rectum. La TDM abdominopelvienne avait montré un processus bourgeonnant hémi-circonférentiel hypodense du moyen rectum sans envahissement locorégional ni à distance. La patiente était opérée par voie médiane, elle a eu une résection antérieure du rectum avec rétablissement immédiat de la continuité. Les suites opératoires étaient simples. L’étude anatomopathologique de la pièce opératoire avait montré une prolifération tumorale assez dense faite de cellules souvent fusiformes à cytoplasme mal délimité et à noyau allongé parfois ovulaire, présentant des atypies légères à modérées et des mitoses nombreuses (20 mitose/50 champs à fort grossissement). L’étude immuno histochimique avait confirmé que les cellules tumorales étaient fortement positives de façon diffuse pour CD 34, C-KIT, et NSE. Ainsi le diagnostic d'une tumeur stromale du moyen rectum à potentiel malin était retenu et la patiente a été mise sous imatinib (GLIVEC^®^) en adjuvant avec rémission complète avec un recul de 5ans.

## Discussion

Les tumeurs stromales gasro-intestinales (GIST) se localisent préférentiellement au niveau de l'estomac (50 à70%) suivie par l'intestin grêle (20 à30%) [2]. La localisation rectale est extrêmement rare et représente uniquement 5% [1, 2]. La survenue de GIST rectale est sporadique dans la grande majorité des cas, mais il existe quelques prédispositions familiales, telles la neurofibromatose de type I [3] et d'exceptionnelles formes familiales [4]. Les symptômes des GIST rectales sont très peu spécifiques, et ne diffèrent pas de ceux des autres tumeurs rectales [5, 6]. Chez notre patient, la rectorragie et la douleur rectale ont constitué les deux principaux signes. Dans les GIST rectales, la rectoscopie repère facilement une tumeur stromale endophytique réalisant une formation arrondie bombant sous une muqueuse normale ou ulcérée. Lorsque la tumeur est à développement exophytique, la paroi rectale en regard peut apparaître simplement rigidifiée posant un problème diagnostic [6], en effet la biopsie endoscopique conventionnelle est généralement négative dans ce cas vu que les GIST sont généralement recouvert par une muqueuse normale et que ces biopsies sont trop superficielles [7]. L’écho endoscopie joue un rôle primordial en matière de GIST rectale, en effet elle permet de visualiser la tumeur qui se présente généralement sous forme d'une masse arrondie ou ovalaire, plus au moins hypoéchogène siégeant dans la quatrième couche (musculeuse) ou la troisième couche (sous-muqueuse) [8]. Elle permet aussi de réaliser des biopsies par forage pour les tumeurs sous muqueuses avec un taux de positivité de 80 à 90% [8]. L’écho endoscopie fournit également quelques éléments pour l'orientation pronostique de la lésion: appréciation de la taille tumorale, de ses contours, d'une éventuelle infiltration de la graisse ou des organes du voisinage ou d'une nécrose centrale [6, 8]. Les signes écho endoscopiques évocateurs de malignité sont: une taille supérieure à 10 cm, la présence d'une nécrose centrale, l'envahissement des organes de voisinage et la présence des zones kystiques intra tumorales [6]. Le scanner abdominopelvien et surtout l'IRM permettent de bien d’étudier les GIST rectales à développement exophytique et de détecter un envahissement des organes de voisinage. Enfin l'examen clé de confirmation diagnostique reste toujours l’étude anatomopathologique avec un complément d’étude immunohistochimique. Lors de la macroscopie, la mesure du diamètre maximal de la tumeur primitive est un paramètre majeur pour l’évaluation du potentiel évolutif [9]. A l'histologie, la densité cellulaire est généralement forte et homogène, des remaniements nécrotiques, œdémateux et/ou hémorragiques sont d'autant plus fréquents que les tumeurs sont de grande taille. Les cellules sont fusiformes dans 70% des cas, avec une architecture le plus souvent fasciculée, évoquant une prolifération musculaire lisse. Plus rarement, les cellules fusiformes se disposent en palissade ou en « bulbe d'oignon » [9]. Pour l’évaluation du risque de récidive, le compte mitotique se fait sur 50 champs au fort grossissement; ce qui n'est généralement possible que sur les pièces opératoires [9]. L’étude immunohistochimique permet de mettre en évidence une expression de KIT par les cellules tumorales dans 95% des cas [10]. L'expression de KIT est, dans la grande majorité des cas, forte et diffuse à l'ensemble des cellules tumorales. Le marquage peut être membranaire, cytoplasmique diffus et/ou cytoplasmique «golgien». Environ 5% des GIST sont négatives pour KIT; le diagnostic nécessite alors la mise en évidence de mutations des gènes KIT ou PDGFRA au sein de l'ADN tumoral [9, 10]. D'autres marqueurs peuvent être utiles pour le diagnostic de GIST rectal. Miettinen et al ont remarqué que l'expression du CD34 est de 92% dans les GIST rectales alors que l'expression de l'AML est de 14% [5]. Dans notre étude, l'examen anatomopathologique de la pièce de biopsie endoscopique a révélé un aspect typique de GIST maligne, avec à l’étude immunohistochimique des cellules tumorales positives pour le c-kit et le CD34.

Les GIST rectales nécessitent une prise en charge en comité multidisciplinaire. Pour les formes résécables, la chirurgicle [11, 12] complète en mono-bloc de la tumeur (résection R0) est le seul traitement curatif des tumeurs stromales digestives. Il est essentiel d’éviter une perforation per-opératoire qui entraîne une dissémination péritonéale et une survie similaire à celle des patients ayant eu une exérèse incomplète dans certaines études [11, 12]. En outre, les énucléations simples sont grevées d'un risque de récidive significativement plus élevé que les résections segmentaires [11]. En cas d'exérèse incomplète (R2) ou d'exérèse de nodules métastatiques péritonéaux associés, le pronostic spontané est mauvais. Le cas des résections R1 reste l′objet de discussions, car il n'a pas été formellement démontré qu'une résection R1 était associée à un moins bon pronostic [11]. Bien qu'il ne soit pas recommandé, Grassi et al. font une excision du méso rectum. Le curage ganglionnaire n'est pas systématique, car les métastases ganglionnaires sont rares et le risque de récidive ganglionnaire est limité [5]. En post opératoire, un traitement à base d'imatinib (GLIVEC^®^) est indiqué pour les formes à risque élevé ou intermédiaire de récidive et discuté en cas de résection incomplète. En effet, à partir des études avec suivi à long terme des patients ayant eu une résection chirurgicale initiale d′un GIST à haut risque concluent que la chirurgie seule n′est généralement pas curative, et que jusqu′à 90% des patients développent des récidives [13]. En outre, dans un essai contrôlé randomisé, Dematteo et al. ont démontré que le risque de récidive des patients sous imatinib (GLIVEC^®^) est significativement moindre par rapport au groupe ayant reçu le placebo [14]. Chez notre patient, ayant un risque élevé de récidive (20 mitoses/50 champs à fort grossissement), une chimiothérapie adjuvante par l'imatinib était indiquée avec bonne évolution.

Concernant les GIST rectales de résécabilité douteuse ou en cas de chirurgie mutilante pour les tumeurs stromales du bas rectum (amputation abdominopérinéale), un traitement néoadjuvant à la base d'imatinib (GLIVEC^®^) est à discuter en unité de concertation pluridisciplinaire. En effet, d'après Hou et al le traitement néoadjuvant à l'imatinib est une option à prendre en compte sérieusement pour éviter un geste chirurgical mutilant tel que l'amputation abdominopérinéale en permettant la préservation du sphincter anal (down staging) et permet de rendre la chirurgie possible pour les tumeurs non résécables [7]. En cas de traitement néoadjuvant, l’évaluation de la réponse au traitement doit être minutieuse. Des explorations précoces avec la PET scan peuvent éventuellement permettre d'identifier un groupe de patients non-répondeurs pour lesquels un geste chirurgical s'impose. Dans la plupart des études, les PET scan ont été pratiquées après un mois de traitement. Les patients répondeurs devraient être opérés dans les 12 mois suivant l'instauration du traitement néoadjuvant [9].

Enfin pour les formes métatatiques, le gold standart reste l'imatinib. En effet, dans les séries historiques la survie globale de ces patients était de l'ordre de 18 à 24 mois, le Glivec^®^ permet d'obtenir une réponse tumorale chez 85% à 90% d'entre eux, avec des survies sans progression de 24 mois et des survies globales supérieures à 36 mois [13, 15]. La réponse objective à l'imatinib se traduit par une survie prolongée et, de façon inattendue, les réponses partielles et la stabilisation de la maladie s'accompagnent d'un bénéfice de survie similaire.

**Figure 3:**
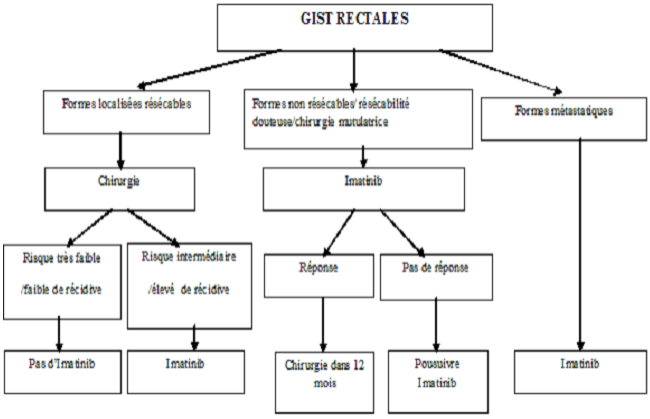
Prise en charge thérapeutique de tumeurs stromales du rectum

On propose l'algorithme suivant dans la prise en charge thérapeutique des tumeurs stromales du rectum (Figure 3)

Le pronostic des GIST rectales demeure réservé: la survie à cinq ans est entre 22 et 66% respectivement pour les tumeurs de haut grade et de bas grade de malignité [12]. D'après «Grassi et al» il y'a tendance de valider les mêmes facteurs pronostiques que les GIST gastriques vu que les GIST rectales sont extrêmement rare et peu étudiés dans la littérature [5].

## Conclusion

Les GIST rectales sont des tumeurs très peu fréquentes. Le diagnostic repose sur l'histologie et l'immunohistochimie. L’échoendoscopie à un intérêt majeur à la fois diagnostique, pronostique et de surveillance. La chirurgie est le traitement de choix pour les formes localisées pour les tumeurs du haut et moyen rectum. La chimiothérapie adjuvante à base d'imatinib (GLIVEC^®^) a amélioré la survie des patients avec GIST rectales localement évolués, métastatiques et des formes à haut risques de récidive. Le traitement néoadjuvant à l'imatinib reste controversé. D′autres études sont nécessaires pour mieux clarifier la stratégie thérapeutique la plus efficace pour les patients atteints de GIST rectale.
